# The Maternal Voice: Exploration of Mothers and Birthing Individuals’ Voices in Patient Safety Event and Feedback Reports

**DOI:** 10.1089/whr.2024.0020

**Published:** 2024-09-26

**Authors:** Allan Fong, Christian L. Boxley, Seth Krevat, Emily K. Mutondo, Angela D. Thomas

**Affiliations:** ^1^Center for Biostatistics, Informatics, and Data Science, MedStar Health Research Institute, Hyattsville, Maryland, USA.; ^2^MedStar Health Research Institute, Hyattsville, Maryland, USA.; ^3^Georgetown University School of Medicine, Reservoir Rd, NW, Washington, DC.

**Keywords:** patient safety, patient voice, incident reports

## Abstract

**Background:**

There is a growing body of research highlighting that Black women have more adverse maternal health events. Instead of only focusing on severe maternal morbidity and mortality events, patient safety events (PSEs) and feedback reports are data sources that can offer insights into a broader spectrum of maternal safety, including near misses, and unsafe conditions. In this work, we explore the racial differences in the representation of mothers and birthing individuals’ (MBIs) voices in PSE and feedback reports.

**Materials and Methods:**

We analyze patient experience themes, dissatisfaction, and disrespect in PSE and patient feedback reports from two large birthing hospitals. We compare racial differences in how the voices of MBIs are represented in these reports. Reports were manually coded for the presence of MBIs’ voices, patient experience themes, dissatisfaction, and disrespect by staff.

**Results:**

In total, 866 reports were reviewed, of which 271 had explicit mentions of MBIs’ voices. A statistically significant association (*p* < 0.001) was observed between patient experience themes and report type, driven by clinical safety event themes in PSE reports. A statistically significant association (*p* = 0.030) was observed between race and report type in 190 reports explicitly mentioning dissatisfaction.

**Discussion:**

Delays with handoff and transfer, pain management, patient staff violence, and procedural harm were proportionally more commonly reported among Black MBIs, supporting prior research in pain management and procedural harm disparities. We also identified themes of positive feedback and good catches by patients, which are key components to patient-centered care and promoting resilience in the care process.

**Conclusions:**

PSE reports tended to capture predominantly clinical themes from Black MBIs, while feedback reports captured predominantly administrative themes from White MBIs. Important perspectives of the safety narrative can be missed when only PSE reports or feedback reports are considered.

## Introduction

There is a growing body of research highlighting that Black women have more adverse maternal health events.^1–10^ Studies on maternal care disparities have found that frequent preventable factors relating to poor maternal health outcomes were provider-related and/or system-related, including substandard providers, delay or failure in diagnosis or recognition of high-risk status, and delay or inappropriate treatment.^1–6^ While these studies are important, they are limited by the narrow focus on severe maternal morbidity and maternal mortality instead of the full spectrum of maternal safety, which includes harm that does not reach the level of severe, near misses, and unsafe conditions.

Patient safety events (PSEs) and feedback reports are data sources that can offer insights into understanding a broader full spectrum of maternal safety.^7,8^ In this study, we explore PSEs and feedback report themes relating to maternal care. We specifically explore racial differences in how the voices of mothers and birthing individuals (MBIs) are represented in PSE and feedback reports as they offer insights into both patient experience and patient safety.

## Background

### Racial disparities in maternal health

There is a growing body of research highlighting that Black women have more adverse maternal health events.^1,2,9,10^ In a Centers for Disease Control and Prevention study, based on analysis of national data on pregnancy-related mortality from 2007 to 2016, Black women were three times more likely to experience maternal harm than White women.^9,10^ Maternal harm is defined as adverse maternal outcomes before delivery, during labor, or after delivery that causes the birthing individual psychological, emotional, or physical harm that is preventable within the walls of the health care system.^9^ For women over age 30, the disparity widened with Black women four to five times more likely to die than white women.^9,10^ The Giving Voice to Mothers Study found that women of color are more likely to experience mistreatment during the perinatal period.^11^ These mistreatment experiences most often included being shouted at, scolded, threatened, ignored, or receiving no response to requests for help.^11^ Black mothers have also reported experiencing stereotypes stigmatizing Black motherhood including assumptions about being single, low income, and having multiple children, which can influence the quality of care delivered.^12^ These types of experiences are important to understand the full spectrum of maternal care but are often missed in traditional outcomes-driven systems that rely on administrative coding and reports by health care staff.

### Patient’s voice

The patient’s voice is a crucial component in identifying potential risks, mitigating harm, and enhancing overall health care quality.^13,14^ MBIs’ voices give insight into their experiences, perspectives, and concerns, which serves as a powerful conduit for understanding the nuances of maternal care and safety. Maternal care is uniquely personal, and a patient’s insights provide invaluable context into the challenges faced during pregnancy, childbirth, and the postpartum period. It is important to recognize that MBIs are not merely recipients of medical care but active participants in their own well-being. Unfortunately, patients’ voices are not always given the attention they deserve within health care systems.^13,14^ This impedes health care systems in providing patient-centered care and the ability of patients and providers to engage in shared decision-making.^11^ It is important to capture and integrate patient voices into patient safety assessments and initiatives to enrich the health care system’s ability to anticipate, identify, and address safety risks, leading to more tailored and effective interventions.

### Importance of PSE and feedback reports

One major shortcoming of the maternal safety literature is its narrow focus on severe maternal morbidity and maternal mortality instead of the full spectrum of maternal safety including precursor events to harm. Patient safety encompasses the systematic identification, analysis, and prevention of errors in health care that can lead to harm. Improving patient safety requires a comprehensive approach that should include analyses of clinical and administrative data sources to uncover patient harm events. These data sources encompass administrative claims data, voluntary event reporting systems, risk management data, patient feedback, and medical record abstraction.^5,15^ PSE reports and feedback reports provide an important lens for connecting MBIs’ voices to the broader understanding of maternal safety and care. Within these data sources, the patient’s voice can emerge as a crucial component in identifying potential risks, mitigating harm, and enhancing overall health care quality.

## Methods

In this work, we explore racial differences in how MBIs’ voices are represented in PSEs and feedback reports. We analyze patient experience themes, dissatisfaction, and disrespect in reports that explicitly mention MBIs’ voices. Patient experience is an overarching term that encompasses the range of interactions that patients have with the health care system.^16^ We leverage the patient experience framework developed through the Consumer Assessment of Healthcare Providers and Systems program.^16^ Patient satisfaction, on the contrary, is about the patient’s expectations about a health encounter. As such, patient dissatisfaction is when a patient’s *expectations* about a health care encounter are not met. Two people who receive the exact same care, but who have different expectations for how that care is supposed to be delivered, can give different levels of satisfaction because of their different expectations.^16^ Patients can be treated with respect or disrespect by staff during a health care encounter.^17^ Respectful treatment of patients by staff involves empathy, emotional support, politeness, dignity, and privacy by the staff. On the contrary, staff exhibiting rudeness, indifferences, and insensitivity are examples of staff disrespect toward patients. We compare the results by the MBIs’ race and discuss the implications and insights of the results.

### Data source

Our dataset consisted of PSE reports and patient feedback reports from two large birthing hospitals in the mid-Atlantic region of the United States between January 1, 2016, and March 31, 2020. PSE reports are voluntary reports by health care staff in which the staff document what they believe to be hazards, safety events, and harm events. Patient feedback reports are collected from patient advocates or directly from patients by conversation, e-mails, phone calls, or survey comments. Reports from both these groups were selected if they involved patients who had a delivery encounter between January 1, 2016, and March 31, 2020. While reports all involve MBIs, the reports themselves might not be directly connected to the birthing experience (e.g., an emergency room visit) but would have to be during a patient’s pregnancy, delivery, or 8 weeks postpartum. Duplicate or non-informative reports (e.g., reports with missing narratives) were removed from the analysis. Patient’s race (Black, White, or Other) was recorded in the electronic health record at patient registration. For this analysis, we focus on Black and White patients, which accounted for more than 80% of the patient population. The various other race categorizations such as Asian and Native American were too small for analysis and were combined into the “Other” category.

### Report coding workflow

**FIG. 1.**
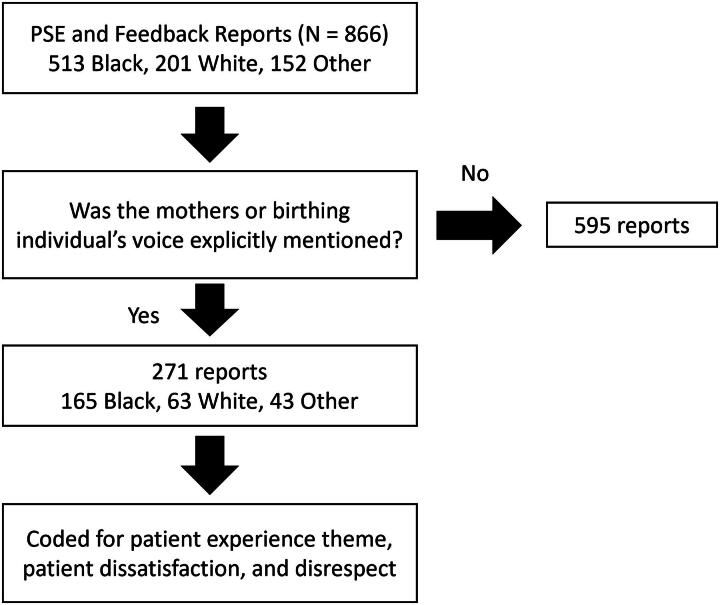
Summary of coding workflows.

For each report, we first identified if the MBIs’ voice was present. To be considered, the MBIs’ voice had to be explicitly mentioned such as quotes from the patient (e.g., “patient states/said/mentioned,” “received a call from patient”) or by a friend or family member. Reports that had MBIs’ voices were further coded for patient experience themes, patient dissatisfaction, or disrespect by staff (Fig. 1). Patient experience themes and sub-themes were iteratively developed by the reviewers. Coding was done by a physician with over two decades of safety leadership and a research analyst with over 5 years of analyzing patient safety reports. The two coders conducted three rounds of separate coding. Each round used a random sample of 90 dually coded reports to establish inter-rater reliability with an average Cohen’s kappa of 0.95. Qualitative coding was done in MS Excel.

### Explicit mentions of patient dissatisfaction

We code reports for explicit dissatisfaction language or tone (i.e., “upset by the treatment,” “not pleased with care,” and “frustrated by staff”) or if a report describes an encounter when a patient’s expectation was not met. For example, “I [the patient] couldn’t get any help with my paperwork…staff told me [the patient] that it is not his job to help with the paperwork, and he is just there to collect the paperwork” indicates a disconnect between the patient’s expectation for help with paperwork and the patient’s experience or not getting help.

### Explicit mention of disrespect

We code reports for explicit mentions of staff disrespect toward a patient (i.e., “[patient] voice her concern regarding the lack of compassion and empathy that the staff showed her,” “[patient] report the rudeness of a nurse towards her,” “[patient] states her RN was rude and unprofessional when she was asking for assistance going to the bathroom”).

### Statistical analysis

We looked specifically at the difference in patient experience themes, explicit mentions of dissatisfaction, and disrespect by race and report type (PSE or feedback). For each categorization, descriptive statistics were calculated. Chi-square tests are used to test for significance between the proportional differences among race and report type for reports with MBIs’ voice. Statistical analysis was done in Python v3.0. This study was approved by the Institutional Review Board at MedStar Health Research Institute (#00005269).

## Results

### Mothers and birthing individuals’ voice

In total, 866 PSE and feedback reports were reviewed. Of these, 271 (31.3% of 866) had explicit mentions of MBIs’ voices. There was no statistically significant difference in the likelihood of reports explicitly mentioning an MBI’s voice and race (*χ*^2^ = 0.819, *p* = 0.66). Of the reports explicitly mentioning the MBIs’ voices, 165 (60.9% of 271) were from Black patients and 63 (23.2% of 271) from White patients (Table 1).

**Table 1.** Proportion of Reports with Mother or Birthing Individuals’ Voice Present Stratified by Race Show No Statistically Significant Differences (*p* = 0.66)

MBI (*N*)	Race				Chi-square	*p*-Value
	Black (513) (%)	White (201) (%)	Other (152) (%)	Total (866) (%)		
Explicit mentions of MBIs’ voices	32	31	28	31	0.819	0.66
Does not explicitly mention	68	69	72	69		

MBIs, mothers and birthing individuals.

For reports with the MBIs’ voices, there was a statistically significant difference between race and report type (*χ*^2^ = 9.05, *p* = 0.011). Proportionally, more Black MBIs’ voices were captured in PSE reports (41.2%), while White MBIs’ voices were proportionally more likely captured in feedback reports (71.4%) (Table 2).

**Table 2.** Proportion of Report Type and Race with Reports Explicitly Mentioning a Mother or Birthing Person’s Voice Shows Statistically Significant Differences (*p* = 0.011)

MBI (*N*)	Race				Chi-square	*p*-Value
	Black (165) (%)	White (63) (%)	Other (43) (%)	Total (271) (%)		
Report type						
PSE report	41	29	19	35	9.05	0.011
Feedback report	59	71	81	65		

PSE, patient safety event.

### Patient experience themes

Five patient experience themes (hospital experience, follow-up care concerns and questions, administrative, clinical safety event type, and other general patient–staff interactions) and 31 sub-themes were identified by the reviewers (Table 3). The complete list of patients experience themes and subthemes is in the Supplementary Appendix SA1, which includes examples and descriptions of each theme. There is a statistically significant relationship between patient experience themes and report type (*χ*^2^ = 104.35, *p* < 0.001). This is driven by the high proportion of clinical safety event themes in PSE reports (59/72, 82%). Five patient experience subthemes occurred most often in PSE reports: 39 out of the 42 (93%) falls reports were PSE reports, 5 out of the 6 (83%) domestic violence reports were PSE reports, 4 out of 5 (80%) medication reaction/errors reports were PSE reports, 4 out of the 7 (57%) delays or issues with handoff/transfer reports were PSE reports, 5 out of the 9 (56%) procedure harm reports were PSE reports. However, the following eight patient experience subthemes occurred more often in feedback reports. All 18 difficulty scheduling/rescheduling reports were feedback reports. All 16 delays in diagnosis or treatment reports were feedback reports. All nine requesting medical records reports were feedback reports. Sixteen out of the 17 (94%) follow-up care questions reports were feedback reports. Seven out of the eight (88%) physical environment issues reports were feedback reports. Nine out of the 11 (82%) lost and found reports were feedback reports. Nine out of the 11 (82%) dietary concerns reports were feedback reports. Nine out of the 11 (82%) conflicts between patient preferences and standard operating procedures reports were feedback reports. Delays with handoff/transfer, pain management, patient staff violence, procedural harm, and patients wanting to smoke in the hospital were themes that were proportionally more commonly reported among Black patients through PSE reports. Translator concerns were also more commonly reported among Black patients and reported more in feedback reports, which could be reflective of the patient demographics. There is no statistically significant relationship between theme categories and race (*χ*^2^ = 3.63, *p* = 0.89). However, proportionally more Black patients tended to have themes relating to general patient–staff interaction and clinical safety event types, 70% and 65%, respectively.

**Table 3.** Aggregated Results of General Themes, Race, Report Type, and Mentions of Dissatisfaction (Dissat.) and Disrespect

Patient experience themes (*N*)	Black (165)	White (63)	Other (43)	PSE (94)	Feedback (177)	Dissat. (190)	Disrespect (53)	Total (271)
Hospital experience (*N*)	38% (62)	40% (25)	37% (16)	29% (27)	43% (76)	45% (85)	47% (25)	38% (103)
Delay in diagnosis/treatment	11%	28%	13%		21%	19%	4%	16%
Delay in discharge	2%				1%	1%		1%
Delay or ignored inpatient room	2%	4%			3%	2%	8%	2%
Delay or issues with handoff/transfer	8%	4%	6%	15%	4%	4%	8%	7%
Dietary concerns	11%		25%	7%	12%	12%	16%	11%
Domestic violence	10%			19%	1%	1%		6%
Pain management	24%	12%	25%	30%	18%	20%	16%	21%
The patient plan was not followed	10%	24%	13%	11%	14%	16%	20%	14%
Patient-staff violence	5%	4%		11%	1%	1%		4%
Physical environment issue	2%	20%	13%	4%	9%	9%	12%	8%
Privacy concerns	8%				7%	6%	12%	5%
Translator	5%			4%	3%	4%		3%
Waiting to be seen in the waiting room	2%	4%	6%		5%	5%	4%	4%
Follow-up care concerns/questions (*N*)	5% (8)	6% (4)	12% (5)	1% (1)	9% (16)	8% (16)	13% (7)	6% (17)
Administrative (*N*)	25% (41)	27% (17)	26% (11)	5% (5)	36% (64)	34% (64)	17% (9)	25% (69)
Billing issue	12%	18%	36%		19%	19%		17%
Difficulty scheduling/rescheduling	24%	29%	27%		28%	28%	56%	26%
Documentation issue/error	12%	12%	9%	60%	8%	8%		12%
Help with paperwork			9%		2%	2%	11%	1%
Hospital policies and procedures	12%				8%	8%	22%	7%
Lost and found	22%	12%		40%	14%	14%		16%
Parking issue	5%		18%		6%	6%	11%	6%
Requesting medical records	10%	29%			14%	14%		13%
Transportation issue	2%				2%	2%		1%
Clinical safety event type (*N*)	28% (47)	24% (15)	23% (10)	63% (59)	7% (13)	8% (15)	9% (5)	27% (72)
Fall	63%	60%	40%	66%	23%	20%	20%	58%
Discharge issue	4%		20%		31%	27%		6%
Medication reaction/error	6%		20%	7%	8%	7%		7%
Other	6%	7%		7%				6%
Procedure harm	17%	27%	10%	15%	31%	33%	60%	18%
Pysch/manic/suicidal	4%	7%	10%	5%	8%	13%	20%	6%
General patient–staff interaction (*N*)	4% (7)	3% (2)	2% (1)	2% (2)	5% (8)	5% (10)	13% (7)	4% (10)
Other	29%	50%	100%		50%	40%	43%	40%
Patient smoking	29%			100%		20%		20%
Reception	42%	50%			50%	40%	57%	40%

Patient experience themes are shown as proportions of the totals based on race, report type, and mentions of dissatisfaction and disrespect. Patient experience subthemes are shown as proportions of their respective main theme.

### Explicit mentions of dissatisfaction

In total, 190 reports explicitly mentioned dissatisfaction. Among these reports, there was a statistically significant relationship between race and report type (*p* = 0.030) (Table 4). Most reports with dissatisfaction were feedback reports (177/190, 93.1%). However, PSE reports with explicit mentions of dissatisfaction were 92.3% (12/13) of Black patients. The most common patient experience subthemes were difficulties scheduling (10/18 Black patients), pain management (11/17 Black patients), delays in diagnosis or treatment (7/16 Black patients), follow-up care questions (7/16 Black patients), and patient plan not followed (6/14 Black patients). It is possible for the same patient experience theme to be expressed without explicit dissatisfaction. For example, 17 out of 22 pain management reports were also coded as explicit mention of dissatisfaction. “Pt medicated with toradol at 1830 and called for pain medication again in 2009. Medicated again by charge nurse for pain” is an example of a pain management theme without explicit mention of dissatisfaction. “MD spoke to the pt over the phone who is now irate and demanding narcotics for pain medications, as Tylenol and Ibuprofen are insufficient for her pain. Pt’s husband is highly upset and feels that no one is listening to his wife and is assuming she is seeking drugs.” This is an example of the pain management theme also coded as explicit mention of dissatisfaction.

**Table 4.** Proportion of Explicit Mentions of Dissatisfaction of Patients in PSE and Feedback Reports Shows Statistically Significant Differences (*p* = 0.030)

Dissatisfaction (*N*)	Black (109) (%)	White (46) (%)	Other (35) (%)	Total (190) (%)
PSE	11	2	0	7
Feedback	89	98	100	93

### Explicit mentions of disrespect

In 53 reports MBIs explicitly mentioned being treated with disrespect by staff. Among these reports, there was no statistically significant relationship between race and report type (*p* = 0.645) (Table 5). Of reports explicitly mentioning disrespect, 56.6% (30/53) were from Black MBIs mostly through feedback reports. The most common subthemes included in reports with explicitly mentioned rudeness were follow-up care questions (3/7 Black patients), scheduling difficulties (2/5 Black patients), dietary concerns (3/4 Black patients), pain management (4/4 Black patients), and reception (3/4 Black patients).

**Table 5.** Proportion of Explicit Mentions of Disrespect of Patients in PSE and Feedback Reports

Disrespect (*N*)	Black (30) (%)	White (12) (%)	Other (11) (%)	Total (53) (%)
PSE	7	8	0	7
Feedback	93	92	100	93

### Additional themes

#### Feeling ignored and leaving against medical advice

Themes of being ignored are presented in multiple ways. This relates to both themes of delays, such as delays in the waiting room, or not having a patient’s plan being followed, such as birthing plans, care, or breastfeeding plans. Sometimes this leads to patients leaving against medical advice (AMA). Six reports concerned four Black patients and two white patients wanting to or leaving AMA due to being ignored, not having a plan followed, and general frustration with the care process.

#### Scared and nervous

In four reports, three Black patients and one other patient explicitly mentioned being scared. These reports centered around an unexpected change in the treatment plan, (“took her off guard and made her feel more nervous”), an unfamiliar experience (i.e., “It was scary to have her baby in the OR”), and lack of effective communication (i.e., “feels this should have been explained to her beforehand”).

#### Good catches

Two reports concerning Black patients had specific mentions of good catches by the patients. These are recorded times when the patient spoke up and it prevented some harm. In the first report, the nurse was about to inject the patient with 100 mg SubQ Lovenox BID when the patient told her she already had the shot that morning. The earlier dose was not recorded in the electronic Medication Administration Record but was later verified in the paper chart. In the second report, the patient alerted the staff to her Motrin allergy which was not documented and prevented an adverse medicine reaction.

#### Positive affirmation of staff

There were also six reports that included positive affirmations or compliments of staff. “There was good education provided on discharge from the post partum unit,” “ED nurses were fantastic,” and “Our experience with the Midwives was excellent… entire delivery went extremely well because of the amazing midwives… felt supported, informed, cared for, safe, secure, and like our concerns were taken seriously” are some examples of compliments and positive affirmations of the staff by the patients. These compliments and affirmations are often interwoven in longer reports.

## Discussion

### Black voices in PSE reports

Delays with handoff and transfer, pain management, patient staff violence, and procedural harm were themes that were proportionally more commonly reported among Black MBIs through PSE reports. This supports other research showing disparities in pain management and procedural harm.^1,2,9,10^ In addition, the tendency for PSE reports to reflect the voices of Black MBIs more often may be correlated to the prevalence of clinical safety event types in PSE reports, especially falls, procedural harm, and medication errors. This might suggest that PSE reports could be more reflective of clinical concerns that might disproportionately affect Black MBIs such as higher clinical rates of maternal mortality and morbidity among Black MBIs in many health care systems. The reporting of patient staff violence is also another interesting theme warranting further investigations into the violence that is happening, how it is perceived, when it is reported, and systems biases that might be present at each step.

### White voices in feedback reports

The tendency for feedback reports to emphasize the voices of white MBIs may reflect a bias in how feedback is collected and considered. Delays in diagnosis and treatments, physical environment concerns, and requesting medical records were themes that were proportionally more commonly reported among White MBIs, especially in feedback reports. This could suggest that proportionally more white patients and families are willing or able to partake in the feedback reporting process, suggesting they might feel more empowered and safer or have tools and resources to provide feedback. This difference could also suggest that the experiences and concerns of white MBIs might be prioritized or more readily acknowledged in the feedback process.

### Implications for practice

This work shows how each data source, PSE reports and feedback reports, provides unique lenses on the overarching topic of maternal safety. PSE reports tended to capture predominantly clinical themes from Black MBIs while feedback reports captured predominantly administrative themes from White MBIs. We also observed that reporters of clinical safety events tended to focus on acute, observable incidents such as falls, which can skew the data toward these types of events. This further underscores the importance of considering the perspectives of both PSE reports and feedback reports in practice as each is critical to the safety narrative. In addition, different patients may have different perceptions of similar events based on expectations suggesting that providers need to not only try to minimize the number of adverse events but must also be sensitive to the different needs and expectations of patients. Lastly, while this work reiterates many previous findings of stereotyping and stigmatizing Black motherhood,^11,12^ we identified times when the MBI’s voices helped to prevent medical error by identifying and alerting health care staff to potential errors before they result in harm. This positive attribution of documenting and recognizing a patient’s good catch should be encouraged in clinical practice as it is a key component to patient-centered care and promoting resilience in the care process.^11^

### Limitations

This analysis is limited by the voluntary and free-form nature of PSE and feedback reports. In addition, we are only considering reports that explicitly mention the patients’ voices. As a result, the free-text narrative is up to the reporter and, even if a patient’s voice is expressed and considered in the care process, it might not have been documented. Although this is a known limitation of this data source, comparing the proportional differences in what gets documented provides insights into biases in the documentation and reporting process. Other patient demographics such as age and marital status were not considered in this analysis but would be important to investigate in further work. Lastly, we do not propose this analysis in isolation as it is important to consider this work in concert with other methods such as interviews, focus groups, and chart reviews.

## Conclusion

We explored patient experience themes in PSEs and feedback reports that explicitly mention MBIs’ voices. The results highlight racial differences in both how MBIs’ voices are captured and the associated patient experience themes. The insights gained from this study contribute another lens to understanding the challenges and opportunities in health care safety and underscore the need for patient-centered approaches in health care settings.

## Supplementary Material

Supplementary Appendix SA1

## Abbreviations Used

BID: twice a day
ED: emergency department
EMAR: electronic medication administration record
MBIs: mothers and birthing individuals
PSE: patient safety event
PT: patient
RN: registered nurse
SubQ: subcutaneous
