# Supplementary material for: The Maternal Voice: Exploration of Mothers and Birthing Individuals’ Voices in Patient Safety Event and Feedback Reports
Source: Womens Health Rep (New Rochelle). 2024 Sep 26;5(1):727–34. doi: 10.1089/whr.2024.0020 (PMC11491569; doi:10.1089/whr.2024.0020)
Supplement: Supplementary Appendix SA1 [file whr.2024.0020_supp_appendix_sa1.pdf]

Appendix 1:

Complete list of patient experience themes and sub-themes in PSE and feedback reports.

**Patient Experience Theme**	**Patient Experience Sub-Theme**	**Examples/Description**
**Hospital Experience**	Waiting to be seen in waiting room	Pt states she routinely waits 45 mins to an hour for her appts in OBGYN clinic after arrival. Pt states she is not notified of why there is a wait and has seen the waiting room turnover several times without any explanation.
	Ignored in patient room	Pt complained the ED care was lacking. Pt provided with a bag for her clothes at check-in and a warm blanket, but pt was left for long periods of time without anyone checking on her.
	Delay or issues with handoff/transfer	Pt was transferred to new room via wheelchair. The pt stated their leg was numb and patient was unable to bear weight.
	Delay in diagnosis/treatment	Pt upset with plan of care during stay. Pt states they have experienced delays with testing and has caused them to remain in the hospital.
	Delay in discharge	Patient stated she is concerned the care team is not effectively working to discharge the patient.
	Dietary concerns	Pt stated the nutrition staff is not taking her orders for meals properly. Pt is improperly given food pt does

		not like.
	Domestic violence	Pt reported that her boyfriend choked her and threatened to kill her when the pt and baby were discharged. The boyfriend was in the room and security arrested him.
	Pain management	Pt should have been given her pain medication in the ED. When pt was asked why she did not receive the medication in the ED, the patient stated the nurse said the medication would be given upstairs. As a result, the patient needlessly suffered for two hours.
	Patient plan not followed	Pt made clear in birth plan that vaginal examinations should be minimized. Doctor was either unaware or did not care. Pt also had to insist not to be examined by a student again
	Patient-staff violence	Staff heard a loud noise coming from pt room. Pt alleged that staff hit her and wanted to report the incident to a supervisor.
	Physical environment issue	Pt fell in front of the elevators when she slipped in a puddle of water. She landed on her extended hand and hip. Pt stated she is still experiencing hip and thumb pain.
	Privacy concerns	Doctor completed rectal exam. Doctor exited and the curtain was left open exposing the patient, who

		wrapped herself in the blanket and closed the curtain. Pt stated they were humiliated, tried to remove her IV, and leave.
	Translator	Pt requires interpreter services for ASL and stated that if there is no interpreter present in the room, she declines all patient care. There is only one interpreter for the hospital at this time.
**Follow up care concerns/questions**	Follow up care questions	Instructions were to f/u with their primary care physician. Pt scheduled appointment but stated they were unsure if they had to go to the appointment. After the pt confirmed that they should be seen, they had many f/u questions and wanted to see if they could be fit into the schedule later the same day.
**Administrative**	Billing issue	Pt contacted advocacy regarding billing for services while she was seen in the ER and inpatient. Patient stated that she is concerned about how much she can afford.
	Difficulty scheduling/rescheduling	Patient communicated that they had a difficult time getting an appointment.
	Documentation issue/error	Patient stated that documentation needed to be improved because their blood pressure was not stable – even though their discharge paper said otherwise.
	Help with paperwork	Pt complained that they were told by staff that it is

		not the staff's job to help with the paperwork – only to collect the paperwork.
	Hospital policies and procedures	Pt's mother voiced confusion regarding the visitation policy for children. Last night, the pt's mother was told that because of the visitation policy, the pt's other children could not stay overnight
	Lost and found	Pt reported lost boots. Patient stated they went missing in labor and delivery and were not transported with pt to postpartum.
	Parking issue	Pt reported their car was damaged in the garage to the point where they could not drive it home from the hospital.
	Requesting medical records	Pt stated she was told that she would receive the medical records within two weeks and stated she did not.
	Transportation issue	Pt asked for assistance in getting a cab voucher home. Pt was instead given bus tokens even though she told the staff that she is not able to use them because of her disability.
**Clinical Safety Event Type**	Fall	Pt was standing and getting ready to be discharged and fell while talking on her cellphone. Pt told the nurse that she did not feel dizzy or lightheaded but was trying to reach down and pick an item up off the

		floor and slipped. Patient is recovering from a c-section.
	Discharge issue including inappropriate discharge (because of a clinical issue) or discharge workflow or process issue	During discharge phone calls, patient stated nurse forgot to take out her PIV. Pt stated she had a nurse she knew personally remove it for her at home.
	Medication reaction/error	Pt allergic to motrin, allergic reactions include hives and itchiness. Motrin ordered for pt. Pt confirmed they have a motrin allergy.
	Other	Pt stated that she was called bi-polar by a staff member.
	Procedure harm	An IV placed in pt's right arm. The contrast had started to infiltrate. The contrast was stopped, and pt was asked if there was pain. Pt stated there was no pain.
	Psych/manic/suicidal	Pt stated that she came to the hospital because she was suicidal. Pt's cousin has agreed for her to come stay with her and does not feel suicidal anymore. Pt discharged.
**Other General Patient-Staff**	Patient smoking	Pt was smoking an electronic cigarette at the bedside. Pt was told she can't smoke, and it is a hospital

**Interaction**		policy because there is oxygen all over the rooms. Pt made clear they were unhappy with the policy.
	Reception	Pt checked into labor & delivery, and pt stated that the front desk staff that she checked in with was rude and unhelpful to her
	Other	Pt feels the seriousness of their surgery was dismissed and ignored. Pt stated the physician's assistant did not listen carefully to their concerns.
